# Supplementary material for: Differences in salivary microbiome among children with tonsillar hypertrophy and/or adenoid hypertrophy
Source: mSystems. 2024 Sep 17;9(10):e00968-24. doi: 10.1128/msystems.00968-24 (PMC11494981; doi:10.1128/msystems.00968-24)
Supplement: Table S1 — The method of determining sample size. [file msystems.00968-24-s0001.docx]

**Supplementary Table 1** Effect sizes (ω^2^) for PERMANOVA power of 80% and 90% with varied sample sizes.

| Sample numbers in each group | ω^2^ / P-value | |
| --- | --- | --- |
|  | power 80% | power 90% |
| 5 | 0.0766 | 0.092 |
| 10 | 0.0311 | 0.0411 |
| 20 | 0.0202 | 0.0269 |
| 30 | 0.00907 | 0.013 |
| 40 | 0.00645 | 0.0105 |

Human Microbiome Project (HMP) dataset was used for distance matrix simulation. “Micropower” package (http://github.com/brendankelly/micropower) was used to assess the effect size and statistical power.
